# Seeing through “brain fog”: neuroimaging assessment and imaging biomarkers for cancer-related cognitive impairments

**DOI:** 10.1186/s40644-024-00797-2

**Published:** 2024-11-18

**Authors:** Quanquan Gu, Liya Wang, Tricia Z. King, Hongbo Chen, Longjiang Zhang, Jianming Ni, Hui Mao

**Affiliations:** 1 Department of Radiology and Imaging Sciences, Emory University School of Medicine, 1750 Haygood Drive NE, Atlanta, Georgia 30322 USA; 2 Department of Radiology, Shenzhen Hyzen Hospital, Shenzhen, 518109 Guangdong People’s Republic of China; 3 School of Nursing, Emory University, Atlanta, Georgia 30322 USA; 4 School of Life and Environmental Sciences, Guilin University of Electronic Technology, Guilin, 541004 Guangxi People’s Republic of China; 5 Department of Radiology, Jinling Hospital, Affiliated Hospital of Medical School, Nanjing University, Nanjing, 210002 Jiangsu People’s Republic of China; 6 Wuxi Second Hospital Affiliated to Nanjing Medical University, Wuxi, 214042 People’s Republic of China

**Keywords:** Cancer survivors, Cognitive impairment, Brain functions, Chemotherapy, Radiation, Neuroimaging, Magnetic resonance imaging, Positron-Emission Tomography

## Abstract

Advances in cancer diagnosis and treatment have substantially improved patient outcomes and survival in recent years. However, up to 75% of cancer patients and survivors, including those with non-central nervous system (non-CNS) cancers, suffer from “brain fog” or impairments in cognitive functions such as attention, memory, learning, and decision-making. While we recognize the impact of cancer-related cognitive impairment (CRCI), we have not fully investigated and understood the causes, mechanisms and interplays of various involving factors. Consequently, there are unmet needs in clinical oncology in assessing the risk of CRCI and managing patients and survivors with this condition in order to make informed treatment decisions and ensure the quality of life for cancer survivors. The state-of-the-art neuroimaging technologies, particularly clinical imaging modalities like magnetic resonance imaging (MRI) and positron emission tomography (PET), have been widely used to study neuroscience questions, including CRCI. However, in-depth applications of these functional and molecular imaging methods in CRCI and their clinical implementation for CRCI management are largely limited. This scoping review provides the current understanding of contributing neurological factors to CRCI and applications of the state-of-the-art multi-modal neuroimaging methods in investigating the functional and structural alterations related to CRCI. Findings from these studies and potential imaging-biomarkers of CRCI that can be used to improve the assessment and characterization of CRCI as well as to predict the risk of CRCI are also highlighted. Emerging issues and perspectives on future development and applications of neuroimaging tools to better understand CRCI and incorporate neuroimaging-based approaches to treatment decisions and patient management are discussed.

## Introduction

Progress in understanding cancer biology, the development of new drugs and targeted therapeutics, and the implementation of personalized care plans have substantially improved treatment efficacy and patient survival. The population of cancer survivors has increased to 18 million in the United States based on the 2022 report and is estimated to reach more than 22 million by 2030 [1]. However, a glaring unmet need is that current patient management and new therapeutic development often overlook and underestimate cancer and/or treatment-associated psychological and neurological impairments in patients across the course of their cancer trajectories, i.e., diagnosis, treatment, recovery, and survival [2]. It is reported that up to 75% of cancer patients experience varying degrees of cancer-related cognitive impairment (CRCI), highlighted with cognitive decline in attention, memory, learning, executive function, and decision-making [3]. These underlying neuropsychological difficulties experienced by cancer patients, commonly referred to as “brain fog” or “chemo-brain”, can disrupt patient adherence to treatment plans and limit their options to different treatments, resulting in decreased efficacy of treatments, lower quality of life during and after treatment, and eventually worse outcomes [3].

Despite such significant burdens in the clinical management of cancer patients and survivors, our understanding of the biological mechanisms of CRCI is still limited. The impact of neurological side effects from systemic treatment, especially chemotherapies, are recognized, but the causes and mechanisms are not well characterized and investigated. Interrogating intertwined contributing factors, requires systematic approaches enabled by tools capable of providing wholistic views of these conditions over the course of disease diagnosis and treatment. Worth noting is that current advancements in anti-cancer drugs and interventions primarily focus on targeting and managing primary tumors, often neglecting the systemic repercussions of treatment on the central nervous system (CNS). This oversight is partly due to the limited ability to determine whether a small amount of therapeutic agents or drugs can reach and accumulate in the brain, subsequently damaging brain tissue and its functions. The systemic treatments commonly used in chemotherapy and, most recently, immunotherapy are especially worrisome when given to patients with diverse health conditions and disease stages. This is because cancer patients, even those with non-central nervous system (non-CNS) cancers, may have a compromised blood brain barrier (BBB) due to comorbidities of pre-existing health conditions and inflammatory responses from treatment side effects [4]. The profound knowledge gap in this area, along with the increasing emphasis on personalized medicine, presents significant challenges in treating and caring for cancer patients while maintaining their quality of life, both during and years after treatment.

Currently, the diagnosis and evaluation of CRCI in oncology clinics primarily relies on self-reported symptoms from patients and sometimes by caregivers. A smaller subset of oncology patients may undergo clinical neuropsychological performance assessments to detect CRCI. Nevertheless, these tests were primarily designed for patients or individuals with neurological diseases or disorders, but not specifically for those with cancer [5].

On the other hand, there have been tremendous advances in the development and implementation of novel neuroimaging technologies in neuroscience research in the past two decades. The state-of-the-art neuroimaging modalities reveal brain structure, physiology, and functions with high spatial resolution, molecular and metabolic information as well as the changes associated with diseases and disorders. However, applications of these imaging tools in CRCI research and patient management are far behind.

In this review, we outline the current understanding on several cancer-associated effects on the brain and cognitive functions of cancer patients, focusing on non-CNS cancers, and their association with patient outcomes. We highlight examples of using clinically adoptable neuroimaging methods, including magnetic resonance imaging (MRI), x-ray computer-assisted tomography (CT), and positron emission tomography (PET), to investigate CRCI. Further applications of the current neuroimaging methods as well as the challenges and future development of this field are discussed, focusing on translating advanced imaging technologies and potential imaging biomarkers for practice-changing clinical applications in oncology.

## Current understanding on contributing factors and mechanisms of CRCI

The cognitive changes and impairments in cancer patients and survivors can be classified into three categories: the effects of cancer itself, patient- and context-related vulnerabilities, and the impacts of therapeutic interventions. The onset and presentation of CRCI vary depending on physical, mental and health conditions of individual patients. Although many conditions can potentially contribute to CRCI, current research efforts and our knowledge on CRCI are focused on several specific areas.

### Age and genetic predisposition

Aging and treatment-related cognitive decline share common mechanisms in cancer patients [6]. It is proposed that cancer and cancer treatment may accelerate normal aging due to increased DNA damage and reduced repair capacity (e.g., shortened telomere length) [6], which limits cognitive reserve and brain reorganization in cancer patients, as observed in older cancer populations [7]. One longitudinal study [8] revealed that older breast cancer patients treated with chemotherapy had poorer baseline cognitive reserve and more severe CRCI, particularly in processing speed, compared to those who did not receive chemotherapy and controls. In addition, age-related hormone level decline has been found to play a role in CRCI as anti-hormonal treatments can amplify hormone fluctuation-induced cognitive dysfunction in elderly cancer patients [9].

Several host germline single nucleotide polymorphisms (SNPs) are associated with CRCI [10, 11], including genes encoding apolipoprotein E (APOE), catechol-o-methyltransferase (COMT), and brain-derived neurotrophic factor (BDNF) [12]. Individuals with these specific SNPs are more susceptible to developing CRCI. Notably, the APOE ε4 genotype is a major risk factor for Alzheimer’s disease (AD) and age-related cognitive deficits. It negatively affects brain functions by altering lipid binding, boosting oxidative stress and inflammation, reducing the metabolism of neural progenitor cells, and damaging the BBB [13]. Patients with these allelic deficits in those alleles have been consistently found to be more vulnerable to CRCI or have poorer cognitive performance [14–17].

### Cancer-induced neuroinflammation and treatment side effects

Most primary cancers are organ-specific, but they also affect overall health of patients, especially the immune system. Emerging evidence has shown that cancer may impair multiple cognitive functions through neuroinflammation [18, 19]. Tumor growth recruits various immune and neoplastic cells into the tissue stroma, increasing the levels of inflammatory cytokines [20]. This mechanism involves upregulated cytokines resulting from cancer-induced systemic inflammation, which can infiltrate the brain via receptor-mediated endocytosis or passive diffusion across the BBB [21]. Elevated cytokine levels activate immune cells in the meningeal and choroid plexus or stimulate inflammatory glial polarization, causing severe biochemical alterations in cognitive-functioning brain structures [22]. In cancer patients, these elevated inflammatory cytokines may disrupt the BBB and impair neural plasticity [23]. Brain-infiltrating immune cells, such as neutrophils [24], monocytes [25], can exacerbate neuroinflammation. A preclinical mouse model of pancreatic cancer cachexia, a condition of cancer-induced weight loss and metabolic disorder, showed that preventing immune cell infiltration into the brain reduces brain inflammation and disease progression, thereby improving cognitive functioning [26].

In general, all chemotherapy medications have been considered neurotoxic [27, 28]. Widely used chemotherapy agents, such as oxaliplatin and cisplatin, can enter both cancer cells and neuronal cells to form mitochondrial DNA (mtDNA) adducts in the brain [29]. Adriamycin or doxorubicin (DOX) can reduce glucose metabolism and cognition by affecting blood vessels in the hippocampus [30]. These medications mainly impact cognitive functions by suppressing neuron activities in the mitochondria [31–33]. Furthermore, combining chemotherapy and adjuvant hormone therapy has been reported to worsen CRCI [34]. A combination of chemotherapy and hormone therapy involving tamoxifen, exemestane, or anastrozole for breast cancer treatment has been linked to impair cognitive performance [35, 36]. Although the exact mechanism is still unclear, an age-dependent effect of tamoxifen on cognition and an endocrine imbalance in hypothalamo-pituitury-adrenal axis caused by decreased estrogen are thought to be contributing factors [37, 38]. Similarly, androgen deprivation therapy (ADT) has been found to negatively affect cognition in prostate cancer patients [39]. Retrospective studies suggest that reduced testosterone following ADT exposure may increase the risk of developing dementia and subsequent AD [40–42].

Targeted therapies and immunotherapies can cause CRCI due to their specific side effects. Recently, checkpoint inhibitors (CPI) targeting immune checkpoint proteins have been associated with CRCI [43, 44]. Although there are only sporadic reports, the underlying mechanism is thought to involve neuroinflammation resulting from changes in proinflammatory cytokine levels and increased microglia activation [45].

### Psychological conditions

Although the exact mechanisms have not been fully defined, psychological disorders and symptoms such as depression, anxiety, insomnia, fatigue, apathy, pain, and general health-related comorbidities have been shown to contribute to CRCI [46–49]. An intriguing finding is that non-CNS cancer patients with lower treatment expectations, upon learning about potential side effects, reported lower self-assessed cognitive function and performed worse in neuropsychological tests [50]. This suggests that cognitive complaints may be more subjective, highlighting the need of more effective psychological interventions in cancer treatment [51–53].

## Neuroimaging of cancer-related cognitive impairments

Various non-invasive imaging modalities, especially clinical imaging modalities in CT, MRI, ultrasound, PET, and integrated PET/MRI, have all been used for studying and visualizing brain structures and changes in normal and disease-affected conditions. These clinical imaging modalities are routinely used by cancer patients in their diagnosis, treatment assessment, and follow-up. Therefore, it is important to leverage the accessibility and capabilities of these imaging tools for investigating cancer related neurological and psychological changes. Given its exquisite soft tissue contrast and 3D deep tissue imaging at sub-millimeter resolution, along with its functional imaging capability, MRI stands out as the most powerful neuroimaging tool for obtaining morphological, physiological, and functional information. In addition, magnetic resonance spectroscopy (MRS) can provide metabolic and neurochemical information associated with cancer-induced alterations. On the other hand, PET provides highly sensitive biomarker-specific molecular imaging and metabolic information that are complementary with MRI and MRS for studying CRCI.

### Cortical and volumetric changes revealed by MRI

**Fig. 1:**
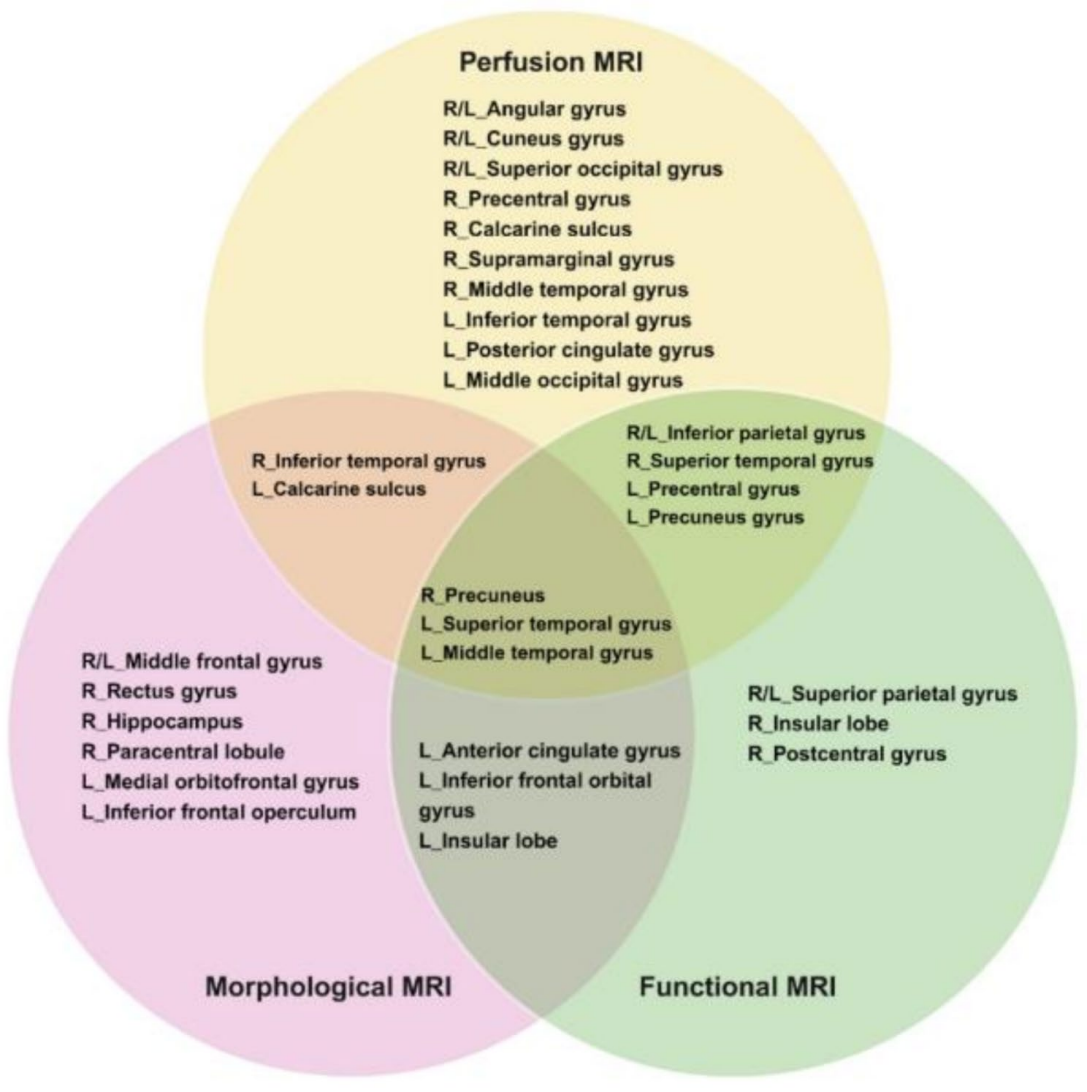
Alterations in Selected Brain Areas in Cancer Survivors. Brain areas that show changes in brain morphology, perfusion and activation after chemotherapy, and several brain areas reveal overlap between modalities. Morphological MRI includes diffusion and anatomical MRI examinations. Functional MRI (fMRI) includes task-related and resting-state fMRI examinations. Perfusion MRI includes arterial spin labelling (ASL) imaging. L = left, R = right Drawn based on the reference by Li & Caeyenberghs [67]. *Created with BioRender.com*

Whole-brain or regional structural alterations in the brain gray matter (GM) and white matter (WM) of cancer survivors after receiving chemotherapy can be quantified using high-resolution 3D T1-weighted imaging with the region of interest (ROI) or voxel-based morphometry (VBM) analysis. Early studies using ROI analysis found that breast cancer patients had a bilateral reduction of the hippocampi volume [54], indicating the high vulnerability of hippocampal structures to chemotherapy [55, 56]. Similar findings have been reported from subsequent VBM studies using high-resolution images to segment specific brain structures [57–65]. The cerebral volumetric loss in cancer survivors after chemotherapy is commonly associated with cognitive impairments such as attention, concentration, verbal memory, executive function, and overall function [58, 59, 62, 64, 65]. While chemo-affected brain areas differ across cancer survivors, significant structural changes are primarily found in the frontal and temporal regions of the brain. Other brain areas have also been identified in previous MRI studies [5, 66] and are illustrated in Fig. 1, including the right middle and superior frontal gyrus, parahippocampal gyrus, bilateral cerebellum, left lateral posterior parietal cortex, bilateral precuneus, left occipital cortex, sub-genual and anterior midcingulate cortex, inferior temporal gyrus, right paracingulate gyrus, left inferior frontal operculum, supramarginal gyrus, temporal area, right amygdala, caudate, and left hippocampal regions.

Studies also found that the chemo-related volumetric changes based on MRI measurements might manifest within a few months (typically one month) after cancer treatment [61, 68], which may persist for months [61, 69] or perhaps extend to years or even decades [62, 70]. These volumetric changes in the brain caused by chemotherapy may undergo a self-compensatory and reversible process [71]. Chemotherapy regimens, genes/SNPs, sex, age, hormone levels, and proinflammatory conditions can also exert such cortical structural change in chemotherapy treated cancer survivors [62, 72, 73].

Analyzing brain volumetric changes using deformation-based morphometry (DBM), another automated morphometric analysis enhanced over the standard VBM, revealed significant widespread WM enlargement and GM reduction in premenopausal breast cancer survivors after receiving chemotherapy for 5 ∼ 6 months [74]. The degree of WM expansion is age-dependent and can be correlated with the level of cognitive decline, linking this form of structural change with a protective mechanism against chemo-related neurotoxicity [74].

In addition to only measuring brain volumetric changes, changes in the cortical thickness and surface area have been found to be more sensitive imaging biomarkers for finding regional cortical deformation associated with aging and diseases. These measurements can be obtained by computing the distance between the WM boundary and the pial surface at each location of the cortex [75]. It is reported that non-CNS cancer survivors have reduced brain surface area and cortical thickness, especially in the frontal and temporal lobes [76]. For example, lung cancer survivors were found to have less cortical thickness in the areas of the frontal, temporal, and insular lobes three to eight months after chemotherapy [72]. Interestingly, although those affected areas are thought to be associated with neuropsychological performance, no statistically significant correlation between those morphological changes and cognitive functions was discovered [72, 77].

In recent years, cortical gyrification, a sensitive imaging marker in the developmental brain, has been used to identify cancer-induced cortical alterations [78, 79]. In a study of older breast cancer survivors 5 to 15 years after chemotherapy, more gyrification has been shown in the right fusiform, paracentral, precuneus, superior, middle temporal gyri, and left pars opercularis, while less gyrification was observed in the left superior parietal gyrus and left cuneus gyrus [80]. The increased right paracentral gyrification was negatively correlated with cognition composite scores for visual vocabulary and oral reading recognition based on past learning experiences [81], suggesting a long-term chemo-related cognitive impairments in older cancer survivors.

To further examine the potential mechanisms underlying the reported correlations between brain structures and cognitive performance in CRCI, more sophisticated graph theory-based network analysis has been used to identify disruptions in brain structural connectivity. It is observed that breast cancer survivors had lower small-world properties after chemotherapy compared to matched healthy controls [82]. Since altered brain topology has been associated with AD [83], breast cancer survivors having a higher risk of developing AD compared to healthy controls and chemotherapy-naïve patients [84], possibly due to declining efficiency and function in both regional and global networks.

### Microscopic changes and disrupted connectivity revealed by diffusion-based MRI

Brain WM consists of a well-organized network of axon fibers or tracts that facilitates structural connections among various cortical areas, which are important for coordination of different cortical structures to perform complex cognitive tasks. Damages in WM tracts can impede neural impulse transmission in the cognitive performance [85]. In breast cancer patients, these changes can manifest as early as three months after treatment and then stabilize six months later [86].

**Fig. 2:**
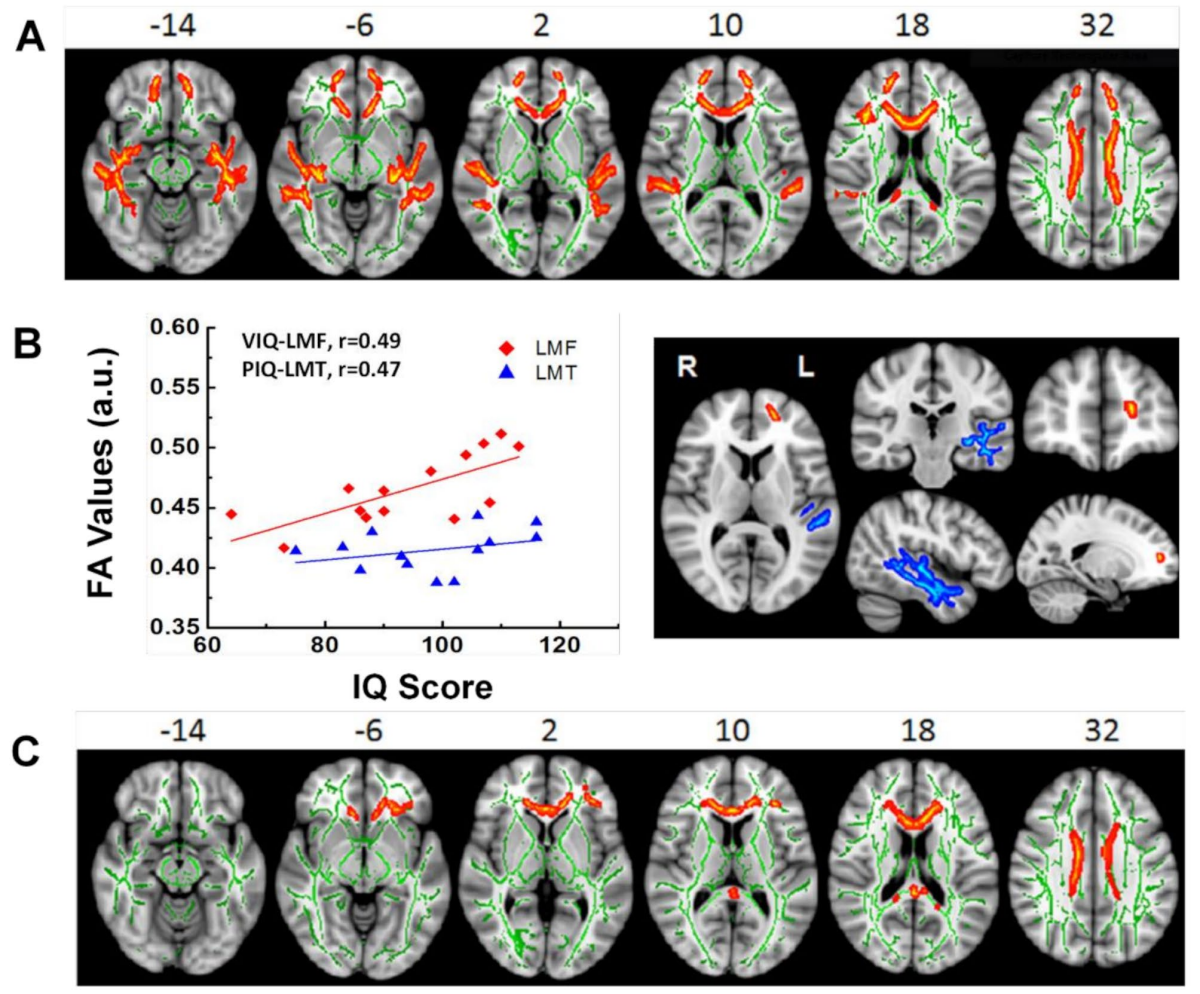
White Matter Changes in Adult Survivors of Childhood Brain Tumors. (**A**) Significant white matter (WM) differences of fractional anisotropy (FA) were found between survivors with and with no radiation treatment and healthy controls (HC). Clusters of significantly lower FA for survivor group were in orange and red. (**B**) The plot of statistically significant correlations between intellectual performance and the WM FA measured from the areas of left frontal pole (LFP) in red, right frontal pole (RFP) in blue and corpus callosum (CC) in green. The open symbols represent survivors with radiation treatment and solid symbol represent those with no radiation treatment. (**C**) Significant WM differences between survivors with no radiation treatment and HC. Clusters of significantly lower FA for survivor group were in orange and red. No statistically significant correlation between intellectual performance and the WM FA was found in these areas. WM skeleton (in green) was overlaid on a T1 weighted image. VIQ = verbal intelligence quotient, PIQ = performance intelligence quotient. a.u. = arbitrary units. R = right, L = left. Adopted from King et al. [88]

While WM hyperintensity on conventional T2-weighted imaging is the imaging feature of WM changes following chemotherapy [87], diffusion weighted imaging (DWI), and particularly diffusion tensor imaging (DTI), are employed to identify and describe the WM alterations at a microscopic level. Fractional anisotropy (FA) and diffusivity measurements (e.g., mean, radial, and axial diffusivity) are the most commonly used parameters derived from DWI and DTI to characterize the structural integrity of WM tracts. Patients who underwent chemotherapy exhibit extensive microscopic WM damages based on the measurements of these parameters [88, 89]. These WM changes mostly observed in the regions of corpus callosum, superior corona radiate, and fibers within the frontal, temporal, parietal, and occipital areas. They are coupled with increased water content within myelin and water diffusivity in axons compared with healthy controls and those cancer patients not receiving chemotherapy [90–92]. As an example shown in Fig. 2, adult individuals who survived childhood cerebellar brain tumors had regionally reduced FA, demonstrating persisting WM impairments years after treatment. These impairments were associated with disrupted cognitive performance that continues into adulthood, even decades after completing treatment during childhood [88, 89]. Although the association between WM microstructure degradation and cognitive dysfunction is well established, the results on the long-term effect over a decade or even decades following chemotherapy remain inconclusive [62, 70, 93, 94], requiring systematical and in-depth investigations in the future.

Additionally, graph theory-based analysis of WM-based structural network analysis has also been applied to investigate changes in structural connectivity in chemo-treated cancer patients to explain impaired memory and executive function in cancer survivors. Compared to healthy controls, breast cancer survivors who underwent chemotherapy exhibited significantly poorer cognitive performance and longer characteristic path lengths, defined as the average shortest path length between all pairs of nodes in the graph of WM tracts [91]. The prolonged path length indicates worse global integration as well as greater segregation within the structural network, which suggests a compensatory mechanism for this brain reorganization.

### Altered activations and functional connectivity observed in BOLD-fMRI

Blood oxygen level-dependent functional MRI (BOLD-fMRI) detects and maps task-invoked activity during a neurophysiological event or resting state brain activation within the whole brain non-invasively. Functional connectivity (FC) analysis is usually used to study the functional coherence of anatomically separate brain regions over the whole brain space. The most popular methods for analyzing FC are the seed-based time series correlation analysis, independent component analysis (ICA), and graph theory-based analysis.

**Fig. 3:**
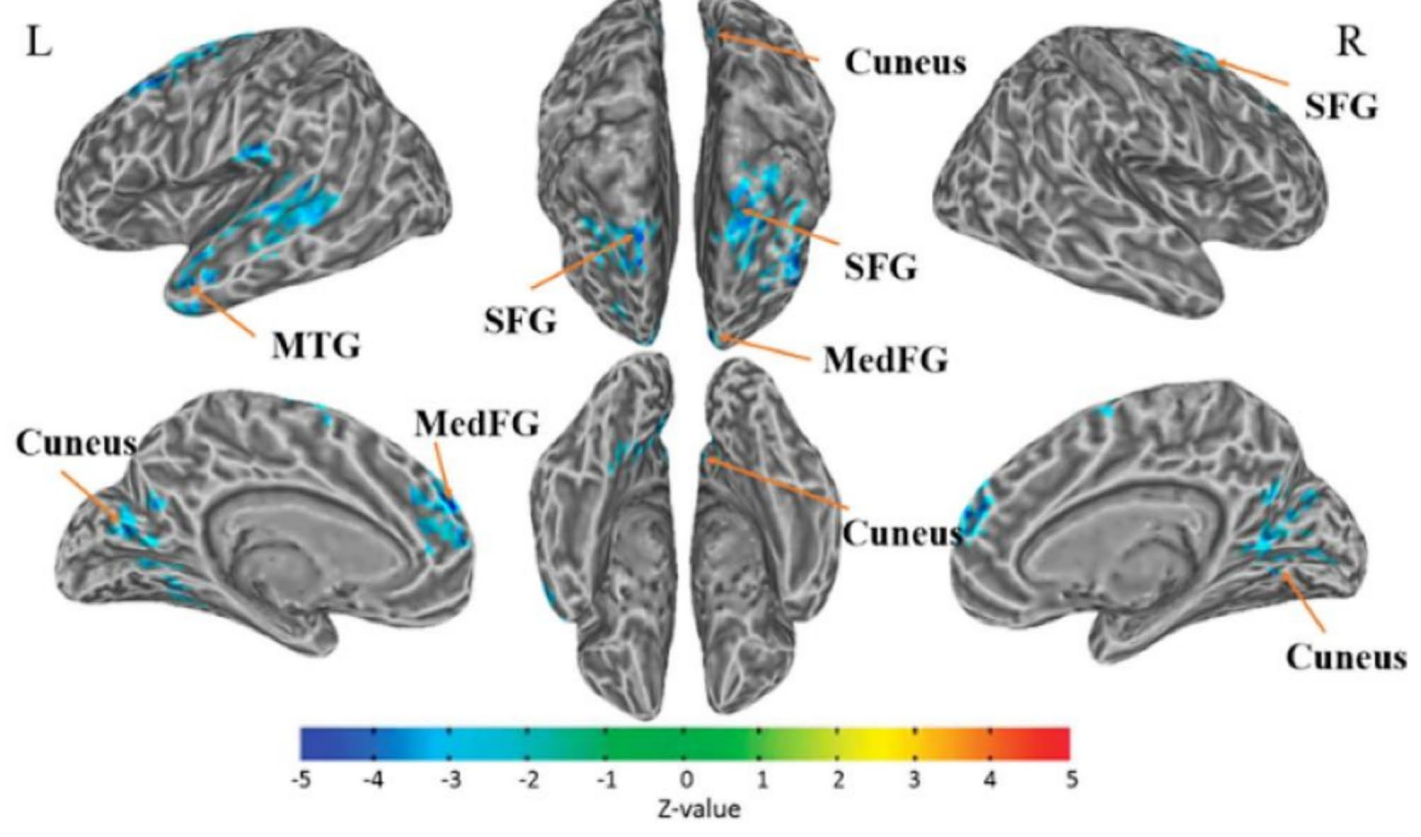
fMRI Detected Functional Connectivity Changes in Cancer Survivors. Demonstration of the lower connectivity areas with the anterior cingulate cortex (ACC) determined by the seed-based method. The color represents the degree of correlation (z-value) between functional connectivity strength. The altered connectivity areas included left superior frontal gyrus (L_SFG), left medial frontal gyrus (L_MedFG), left middle temporal gyrus (L_MTG), left cuneus, right cuneus, and right superior frontal gyrus (R_SFG). L = left hemisphere; R = right hemisphere. The threshold was set to *P* ≤ 0.05 (corrected with the Monte Carlo method). Adopted from Miao, et al. [103]

The first fMRI study on CRCI was conducted by Ferguson and colleagues [95]. They compared the activation map of working memory between a breast cancer patient 22 months after chemotherapy and her monozygotic twin sister. They found the cancer patient with CRCI exhibited increased levels of activation in the frontal and parietal regions, which was explained by the compensatory recruitment of a broader neural network to accomplish cognitive performance comparable to that of her unaffected twin. However, more cross-sectional [68, 96] and longitudinal [97] studies comparing pre- and post-treatment showed chemo-treated survivors with rather suppressed activation in regions, such as the hippocampus, precuneus, cingulate gyrus, prefrontal, premotor, and temporal cortices as shown in Fig. 3. These functional alterations are usually accompanied by worse cognitive performance [98, 99]. The reduced activation was interpreted as: (1) diminished baseline functional activation patterns caused by cancer and treatment [100]; (2) insufficient compensation for the functional damage after chemotherapy [101]; and (3) physical and psychological stresses when coping with the disease, which accelerate or worsen these cognitive dysfunctions [102].

Regarding functional networks, FC analysis showed reduced connectivity in regions within the default mode network (DMN) [104], central executive network (CEN) [103, 105], and salience network (SN) [105] in chemo-treated cancer survivors [106, 107]. Within the DMN, altered functional activation in the precuneus has been linked to the types of cognitive impairments in CRCI, including episodic memory retrieval, visuospatial imagery, and self-referential processing [98]. The possible reason is that the functional centrality and higher metabolic demands during brain activation may render the precuneus less resistant to diseases, aging processes [108], and chemotherapy neurotoxicity [109, 110]. Another study on chemo-treated multiple myeloma patients [105] found that cognition-related frontal-parietal regions within the CEN and SN were more vulnerable to chemo-induced neurotoxicity. Furthermore, a large-scale FC analysis of breast cancer patients [111] revealed that altered FC between DMN and other subnetworks may be more sensitive to cognitive changes in the chemo-treated patients, suggesting the importance of evaluating integration and segregation of functional networks in assessing patients for possible CRCI.

In order to find cancer-related changes in topological features of the neural network such as small-worldness, modularity, centrality, and regional network parameters, the graph theory-based brain network analysis is commonly used [112]. This approach defines vertices (“nodes”) as brain regions, and connections (“edges”) as structural and functional connectivity between regions to evaluate brain network efficiency and organization based on fMRI data [113]. In this case, small-world networks have the shortest path between pairs of nodes and the fewest edges, representing high efficiency, optimized organization, and stable topology for signal processing [114, 115]. The parameter of hubness refers to nodes with high nodal centrality interconnecting nodes between different modules or in the same module extensively [116]. It is found that chemo-treated breast cancer patients have compromised small-worldness and network efficiency in the frontotemporal areas [82]. Interestingly, these functional network changes resemble those observed in AD patients [83], suggesting the higher risk of developing cognitive dysfunction or AD related dementia in those chemotherapy-treated patients compared to chemotherapy-naïve patients and healthy controls [84]. Similarly, reduced nodal and global network efficiency have been reported in breast cancer survivors with cognitive deficits five years after chemotherapy [117].

Collectively, changes in functional networks, along with structural connectivity, can serve as an indicator for early diagnosis and prediction of CRCI. Similar to the recovery of structural changes, regional FC usually can recover, in some extents, one year after chemotherapy [77, 100]. In breast cancer patients, FC recovery can be seen as early as one week after chemotherapy, which continues further six months later and even returns to baseline levels eventually [111]. However, self-reported memory deficits arise a month to one year later [61], suggesting that CRCI-related brain network changes appear late in the development of CRCI. Moreover, a study on network-based topological changes in breast cancer patients demonstrated that pre-existing neurological deficits made individuals more vulnerable to the effects of chemotherapy, other adjuvant treatment and even normal aging [118]. Therefore, it is important for oncologists to consider these new information and conditions to select proper treatments with low risks of causing CRCI.

### Molecular and metabolic imaging of neurochemical changes

**Fig. 4:**
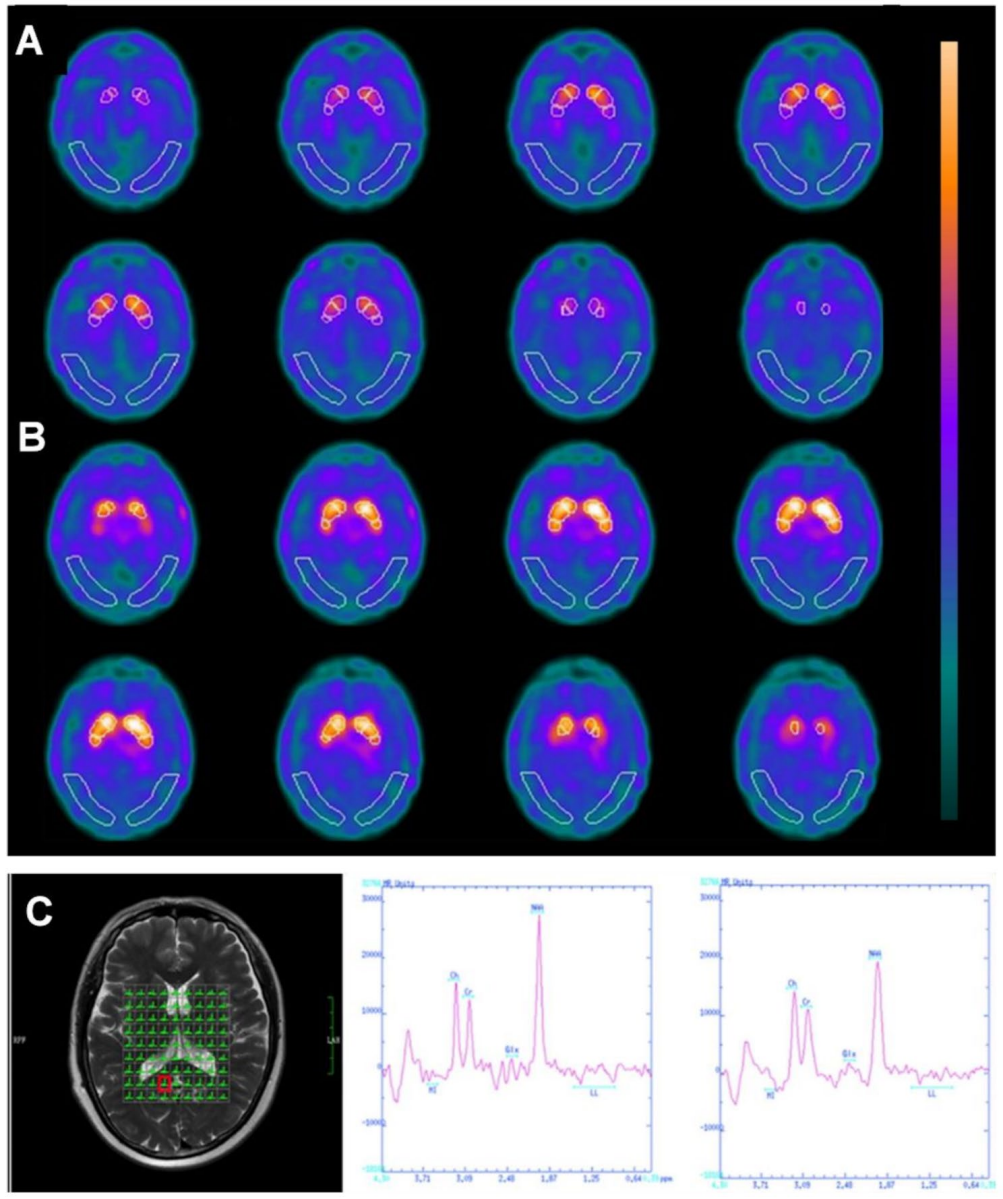
Metabolic Changes Shown in PET and MRS. Volumes of interest (VOIs) superimposed on images of a 62-year-old chemobrain patient received TRODAT-1 scan (**A**) and an age-matched control subject (**B**). Target region VOIs include the entire striatum, head of caudate nucleus, entire putamen, anterior putamen, and posterior putamen. Compared to the paired control (**B**), patients’ images (**A**) show decreased striatal uptake. Adopted from Vitor et al. [125]. (**C**) an example of the volumes of interest for multivoxel magnetic resonance spectroscopy (MRS) (Left) and the attained spectra (Middle and Right) from a breast cancer patient. The region of interest was placed in the right posterior cingulate cortex, and significantly lower NAA values were shown after treatment (Right) compared with before chemotherapy (Middle). Adopted from Tong et al. [126]

Neuroinflammation and neurotoxicity caused by cancer and chemotherapy can affect neurochemistry and brain metabolism, which can be investigated by various non-invasive and clinically available oncology imaging methods like PET, single-photon emission computed tomography (SPECT), and MRS [119] (Fig. 4). However, biochemical and metabolic alterations in CRCI are understudied compared to structural and functional changes reported from aforementioned MRI studies, thus presenting opportunities for future studies and clinical applications.

SPECT and PET are the most prevalent nuclear medicine techniques that use radioactive tracers or substrates to examine tissue metabolism and neurological function before and after chemotherapy [120]. 99m-Technetium-hexamethyl propylene amine oxime (^99m^Tc-HMPAO) and ^99m^Tc-labeled tropane derivative (^99m^Tc-TRODAT-1) are SPECT tracers that have been used for studying CRCI. The former is a neutral lipophilic agent used to measure cerebral blood flow [121], while the latter is a dopamine transporter (DAT) radioligand applied to study neuronal degeneration [122]. ^99m^Tc-HMPAO SPECT imaging enables the detection of chemotherapy-induced brain microvascular damage [123] and identifies diffusely heterogeneous hypoperfusion as an early imaging indication of neurotoxicity in children with leukemia [124]. Using ^99m^Tc-TRODAT-1 imaging, a significantly reduced striatal uptake was found in breast cancer survivors with CRCI, suggesting CRCI-related neurotoxicity specific to the basal ganglia [125].

One of the most recognized physiological features of the brain is the high level of glucose metabolism driven by synaptic activity. However, post-chemotherapy lymphoma patients with CRCI have been shown to have reduced 18F-fluorodeoxyglucose (^18^F-FDG) uptake in cognition-related areas of prefrontal cortices, cerebellum, medial cortices, and limbic areas at resting state [127] or during performing tasks [128]. The observed reduction in glucose metabolism was negatively correlated with chemotherapy cycles and positively correlated with post-treatment time [129]. To study neuroinflammation in CRCI, probes targeting the translocator protein (TSPO) have been used [130]. TSPO is a transmembrane protein in the mitochondrial membrane that is expressed at a low level in healthy brains but substantially overexpressed when microglia are activated in neuroinflammation. Using TSPO targeted ^18^F-labeled N, N-diethyl-2-(2-[4-(2-fluoroethoxy)phenyl]-5,7-dimethylpyrazolo[1,5-α]pyrimidine-3-yl)acetamide (^18^F-DPA-714), PET-MR imaging showed that patients treated with epirubicin combined with cyclophosphamide and paclitaxel had a higher probe uptake than chemotherapy-naïve ones and healthy controls, revealing chemotherapy caused neuroinflammation [131].

The concentration of neuronal metabolites such as N-acetyl aspartate (NAA), creatine (Cr), choline (Cho), glutamate, myo-inositol (mI), lactate, and GABA can be measured by MRS on most clinical MRI scanners [132]. The lower NAA concentration in the disease-affected brain indicates losing neurons resulting from degenerations, injuries, or other damages, which can lead to reduced neuronal activity or malfunction [132]. mI is a precursor of inositol triphosphate and phosphatidylinositol, serving neuronal excitability and neurotransmitter release [133], which strongly correlates to neuroinflammation [134]. Moreover, the increase in the Cho level is an indicator of increased membrane turnover when cell membranes break down rapidly, as seen in aggressively growing tumors with high cellularity and CNS neuroinflammation [119, 135]. Breast cancer survivors exhibited elevated levels of Cho and mI in the brain after chemotherapy, which were correlated to reduced executive function and memory ability [136]. The possible explanation is that increased mI is increased to reduce inflammatory effects mediated by cytokines [137], while the increased Cho may result from the compensatory release of free choline for acetylcholine deficiency in membrane maintenance [138].

### Imaging of dysfunctions in BBB and the glymphatic system

BBB is a complex structural and functional barrier composed of endothelial cells, pericytes, the basement membrane, and the astrocyte end-foot [139]. The normal BBB keeps most macromolecules and neurotoxins out of the CNS and maintains steady brain uptake of nutrition and regulatory molecules while protecting the brain and its environment from exposure to harmful substances. Systemic inflammation due to development of cancer and chemo-, radiation treatment can increase BBB permeability by disrupting BBB components and functions, allowing more chemotherapy compounds to reach the brains of cancer patients. However, the precise impact of cancer development on the BBB, as well as the specific types and treatment methods, remain unclear.

**Fig. 5:**
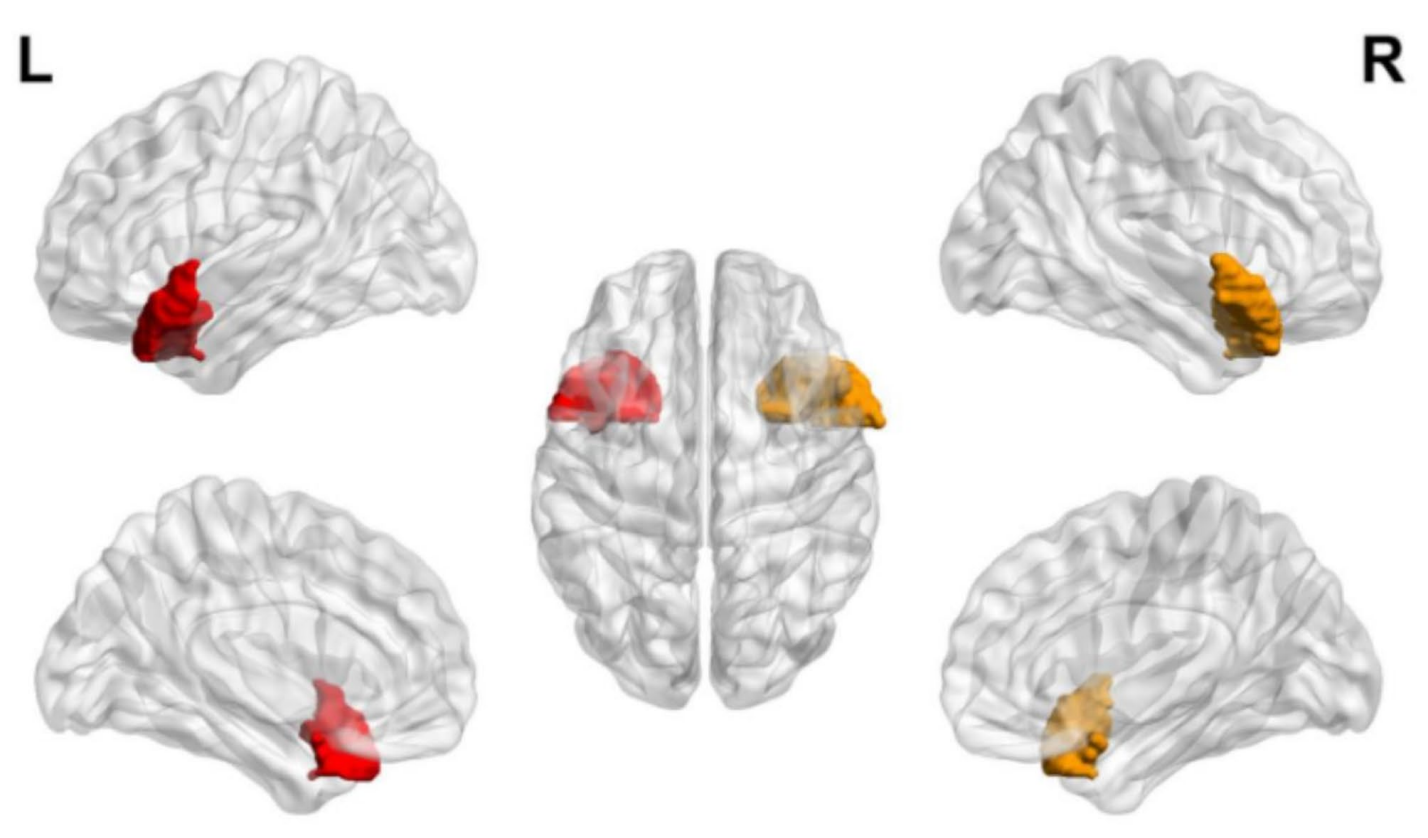
DCE-MRI Detected BBB Disruption in Lung Cancer Patients without Brain Metastases. Compared with healthy controls, the Ktrans of bilateral temporal gyrus were increased in lung cancer patients, mainly in patients with advanced lung cancer (*P* = 0.042). Adopted from Zhang et al. [143]

Increasing efforts have been made to non-invasively investigate the events and processes of BBB disruption using various modalities, including PET, dynamic contrast-enhanced MRI (DCE-MRI) with injection of a contrast agent, and arterial spin labeling (ASL) without using exogenous contrast agents [140] but using magnetically labeled arterial blood water protons as an endogenous tracer instead of injected exogenous contrast agents. PET assesses BBB function by measuring brain uptake of PET tracers of ^11^C-butanol and ^15^O-H_2_O. It has higher sensitivity, spatial resolution, and fast scan time. However, using imaging tracers with an extremely short half-life (i.e., ^15^O, 2 min; ^11^C, 20 min) limits its use in oncology clinics for assessing BBB and CRCI [141]. On the other hand, DCE or DSC MRI using blood-pool small molecule contrast agents is commonly performed on cancer patients for diagnosis and treatment monitoring. Thus, there is a growing interest and technical development effort in leveraging DCE or DSC MRI for studying BBB integrity. The parameters used for describing BBB permeability are the volume transfer constant of K_trans_ obtained from analyzing DCE-MRI data and the water exchange (K_w_) calculated from ASL [141]. DCE-MRI uses gadolinium-induced signal changes when blood crosses the BBB to quantify BBB permeability but is insensitive to minor and early BBB dysfunction [142]. Based on DCE-MRI-derived K_trans_, increased BBB permeability has been found in patients with advanced lung cancer without CNS metastases [4]. Moreover, observed BBB damage, as shown in Fig. 5, was associated with cognitive dysfunction of visuospatial and executive functions, and delayed recall [143]. ASL imaging is normally used for measuring blood flow in studying brain perfusion in different conditions and brain activations when blood flow increases in the activated regions. However, it can be also used to study water exchange across BBB. ASL imaging is more sensitive to minor BBB damage than DCE or DSC MRI but with lower signal-to-noise ratio [141, 144]. ASL imaging was shown to be able to reveal compensatory hyperperfusion early in chemo-treated breast cancer patients after receiving chemotherapy. The high water change rate across BBB evidenced by hyperperfusion is correlated to worse pre-treatment neuropsychological performance, suggesting chemotherapy-induced damage preferentially occurring in regions with less cognitive reserve [145].

More recently, the glymphatic system, a unique system of perivascular channels made up of astroglial cells, was discovered and shown to be responsible for waste removal from the CNS, particularly the brain. In the context of CRCI, the structure and functions of glymphatic system, as well as their changes during the courses of cancer development and treatment are new subjects of investigation for their possible roles in managing neurotoxicity and inflammatory responses induced by cancer treatment, especially chemotherapy. The impaired glymphatic system may impede the delivery of hazardous chemical waste, leading to long-term neurotoxicity and cognitive impairment in cancer patients [141].

## Perspectives and conclusion

As new discoveries and advanced cancer therapies continue to improve the outcome of cancer treatment, ensuring the good quality of life for cancer survivors becomes increasingly important. However, our understanding of CRCI and approaches towards managing such conditions are still limited. The state-of-the-art neuroimaging modalities are well suited for studying CRCI in a variety of cancer populations with opportunities to expand their applications and implementation in radiology practices in the multidisciplinary care of cancer patients. In this context, their advantages and limitations need to be considered during development of novel applications for specific biological and physiological variables involved in CRCI.

Emerging evidence has demonstrated the essential roles of systemic inflammation and neurotoxicity, individual health conditions, treatment side effects, and cognitive dysfunction in alterations in brain structure, function, and metabolism of cancer patients and survivors. Various health comorbidities need to be examined for their possible roles in the development of cognitive dysfunction. Breast cancer patients with cardiovascular disease or diabetes have been found to show a higher rate of cognitive impairments than controls before and after systemic treatment [91]. It is recognized that chronic inflammation during cancer development prior to and during treatment can contribute to CRCI [32]. However, the investigation and understanding of the underlying mechanisms need to be carried out in conjunction with studying the natural history of the chronic conditions and the specific treatment applied to the individual patients for their chronic disease and cancer. Given that many cancers are more prevalent in older populations, it is of great interest to study the CRCI along with aging processes and aging-related diseases and disorders that affect these populations. It is likely that the aging-related diseases and disorders can accelerate the development and progression of CRCI and vice versa. The disease mechanisms and comorbidities shared by dementia, cancer, and aging should be considered and investigated systematically with rigorous multimodal quantitative approaches and multidisciplinary collaborations.

Future studies in CRCI should also focus on how emerging new treatments and drugs may affect patients neurologically in terms of both short- and long-term outcomes. With the increased use and success of biomarker-targeted treatments and immunotherapies, more patients will benefit from these practice-changing interventions. However, their side effects and risk of causing CRCI need to be better understood together with our improvement in understanding of neurobiology, physiology, and functions, such as the properties and changes of the BBB and glymphatic system under different conditions. Understandably, due to current limitations in proper tools and methods for in-depth investigations, there is very little knowledge and data on whether they can be affected by cancer and cancer treatment.

Furthermore, it is crucial that CRCI among cancer survivors be investigated and managed within the specific resources and experiences of patients and survivors. There are diverse ranges and types of variables that need to be taken into account, such as challenges to healthcare and basic life resources (clean air, water, housing, and energy), quality and level of education, socioeconomic status, medical insurance status, language barriers, including race or ethnicity, and social behavior and attitude towards specific demographic groups [146]. Until now, these factors and their confounding effects have rarely been considered in CRCI studies, and there have been recent calls to action to better understand social determinants of health when examining cancer outcomes [147]. Future studies including these essential variables are necessary, but the complexity and dynamic nature of social determinants of health must be taken into account at the forefront of neuroimaging literature as well. This is particularly important when considering current studies and the methods used for recruitment, the wide variety of clinical assessment tools employed for CRCI, and the limited representative normative data available for the measures despite a growing diverse and multiracial/ethnic/language society.

As we recognize the great need for better understanding complex and inter-related contributing conditions in CRCI and subjective, repeatable, and quantitative measurements of CRCI, it becomes apparent that the current available cancer imaging modalities in oncology are well poised to make a valuable impact in this area. The future development of neuroimaging methods for CRCI should be first focused on: (1) developing robust imaging protocols, especially with MRI-based approaches, such as DCE-MRI, DTI, and MRS, that can be integrated into the imaging studies in patient standard care; (2) developing a toolbox or system that uses neuroimaging measurements of CRCI with improved performance, i.e., sensitivity and specificity for diagnosis of CRCI with demonstrated validity compared to those in current clinical practice; (3) taking advantage of multimodal imaging capabilities of mapping brain structures and functions, especially PET and MRI, to cover the broad range of biological and psychological mechanisms underlying CRCI. Ultimately, building optimized multimodal neuroimaging with data fusion models for predicting CRCI at the individual level will enhance the standard of care of cancer patients and survivors.

Naturally, imaging methods can also be applied to enhance preclinical studies of the neurotoxicity and pharmacokinetics of new chemo- or immunological drugs and other novel therapeutics, especially in the context of their effects on the brain. For example, emerging new methods for BBB imaging will enable investigating BBB permeability and/or dysfunctional transcellular transport in the conditions associated with CRCI, although less attention has been given to this field currently. Thus, innovative imaging approaches with comprehensive and longitudinal research designs will help understand the dynamic progression of CRCI and establish neuroimaging-guided or informed treatment decision and clinical management in order to make a practice-changing impact on patient care in the future.

## Data Availability

No datasets were generated or analysed during the current study.

## Abbreviations

CRCI: Cancer-related cognitive impairments
MRI: Magnetic resonance imaging
PET: Positron emission tomography
BBB: Blood brain barrier
CNS: Central neurological system
SNP: Single nucleotide polymorphism
APOE: Apolipoprotein E
COMT: Catechol-O-methyltransferase
BDNF: Brain-derived neurotrophic factor
AD: Alzheimer disease
GABA: Gamma-aminobutyric acid
mtDNA: The mitochondrial DNA
ROS: Reactive oxygen species
DOX: Doxorubicin
ADT: Androgen deprivation therapy
CPI: Checkpoint inhibitor
VEGF: Vascular endothelial growth factor
KPS: Karnofsky performance status
PROMIS: Patient-reported outcomes measurement information system
ICCTF: International cancer and cognition task force
MRS: Magnetic resonance spectroscopy
GM: Grey matter
WM: White matter
ROI: Region of interest
VBM: Voxel-based morphometry
fMRI: Functional magnetic resonance imaging
DCE-MRI: Dynamic contrast-enhanced magnetic resonance imaging
ASL: Arterial spin labelling
DBM: Deformation-based morphometry
DWI: Diffusion weighted imaging
DTI: Diffusion tensor imaging
ADC: Apparent diffusion constant
FA: Fractional anisotropy
HC: Healthy controls
BOLD-fMRI: Blood oxygen level dependent functional MRI
FC: Functional connectivity
ICA: Independent component analysis
DMN: Default mode network
CEN: Central executive network
SN: Salience network
SPECT: Single-photon emission computed tomography
^99m^Tc-HMPAO: Technetium-99m hexamethyl propylene amine oxime:
^99m^Tc-TRODAT-1: Technetium-99m-labeled tropane derivative
DAT: Dopamine transporter
^18^F-FDG: 18 F-fluorodeoxyglucose
TSPO: Translocator protein
^18^F-DPA-714: 18 F-labeled N, N-diethyl-2-(2-[4-(2-fluoroethoxy) phenyl]-5,7-dimethylpyrazolo[1,5-α] pyrimidine-3-yl) acetamide)
NAA: N-acetyl aspartate
Cr: Creatine
Cho: Choline
mI: Myo-inositol
